# Targeting hexokinase 2 increases the sensitivity of oxaliplatin by Twist1 in colorectal cancer

**DOI:** 10.1111/jcmm.16842

**Published:** 2021-08-10

**Authors:** Bo Zhang, Sze‐Hoi Chan, Xue‐Qi Liu, Yuan‐Yuan Shi, Zhao‐Xia Dong, Xin‐Rong Shao, Li‐Yuan Zheng, Zhi‐Ying Mai, Tian‐Liang Fang, Li‐Zhi Deng, Di‐Sheng Zhou, Shu‐Na Chen, Miao Li, Xing‐Ding Zhang

**Affiliations:** ^1^ Molecular Cancer Research Center, School of Medicine Sixth Affiliated Hospital Sun Yat‐Sen University Guangzhou China; ^2^ Department of Hematology The First Affiliated Hospital of Shenzhen University Shenzhen Second People’s Hospital Shenzhen China

**Keywords:** chemoresistance, colorectal cancer, hexokinase 2, Twist1

## Abstract

Colorectal cancer (CRC) is the third most malignant tumour worldwide, with high mortality and recurrence. Chemoresistance is one of the main factors leading to metastasis and poor prognosis in advanced CRC patients. By analysing the Gene Expression Omnibus data set, we found higher hexokinase 2 (HK2) expression levels in patients with metastatic CRC than in those with primary CRC. Moreover, we observed higher enrichment in oxaliplatin resistance‐related gene sets in metastatic CRC than in primary CRC. However, the underlying relationship has not yet been elucidated. In our study, HK2 expression was significantly elevated in CRC patients. Gene set enrichment analysis (GSEA) revealed multi‐drug resistance and epithelial‐mesenchymal transition (EMT) pathways related to high HK2 expression. Our results showed that knockdown of HK2 significantly inhibited vimentin and Twist1 expression and promoted TJP1 and E‐cadherin expression in CRC cells. Additionally, transcriptional and enzymatic inhibition of HK2 by 3‐bromopyruvate (3‐bp) impaired oxaliplatin resistance *in vitro* and *in vivo*. Mechanistically, HK2 interacts with and stabilized Twist1 by preventing its ubiquitin‐mediated degradation, which is related to oxaliplatin resistance, in CRC cells. Overexpression of Twist1 reduced the apoptosis rate by HK2 knockdown in CRC cells. Collectively, we discovered that HK2 is a crucial regulator that mediates oxaliplatin resistance through Twist1. These findings identify HK2 and Twist1 as promising drug targets for CRC chemoresistance.

## 1 INTRODUCTION

Colorectal cancer (CRC) is the third most common malignant tumour worldwide and has high metastasis and recurrence rates.^1^ Approximately 60% of CRC patients develop liver metastasis, which is the major cause of CRC‐related mortality.^2^ Although chemotherapy has been developed for patients with advanced disease, intrinsic or acquired resistance to oxaliplatin‐based combinations is still the major cause of treatment failure.^3^ Therefore, it is essential to investigate the chemoresistance mechanism to improve CRC treatment.

Reprogramming of metabolism is acquired by tumour cells in the processes of tumorigenesis and metastasis.^4^ Alterations in glycolysis, glutamine decomposition and fatty acid synthesis make them potential targets for cancer therapy.^5^ Hexokinase (HK), a family of rate‐limiting enzymes in glycolysis, is a hallmark of cancer.^6^ Among the four isoforms of hexokinase, increased expression of HK3 has been reported in colorectal, lung, gastrointestinal and breast cancers.^7^ HK1 is an effector of KRAS4A, which enhances glucose transport and glycolysis.^8^ HK2 deletion in lung cancer cells suppresses glucose‐derived ribonucleotide synthesis and impairs glutamine‐derived carbon utilization in anaplerosis.^9^ In addition to glycolysis modulation, HK2 is considered to regulate cancer progression by mediating intracellular apoptosis signalling cascades.^10^ HK1 is ubiquitously expressed and is the main hexokinase expressed in most normal adult tissues,^6^ while HK2 is highly expressed in many cancers and constitutes the predominant hexokinase in only a limited number of adult tissues.^11^

Twist family bHLH transcription factor 1 (Twist1) is widely overexpressed in multiple human malignancies and promotes the epithelial‐mesenchymal transition (EMT) process through several signalling pathways. The EMT process modulates immune escape and therapeutic resistance by promoting homologous recombination‐mediated DNA damage repair and the resulting resolution of DNA double‐stranded breaks.^12^, ^13^ Cells that have undergone a partial EMT programme exhibit heightened resistance to apoptosis or the ability to extrude cytotoxic drugs.^14^, ^15^ It has been reported that the FBXW7‐ZEB2 and Twist1‐ABCB1 axes promote chemoresistance in CRC.^16^, ^17^ Slug/MAPK‐mediated regulation of cisplatin resistance has been detected in ovarian cancers.^18^ It has been found that upregulation of HK2 in a hypoxic environment can promote EMT in tongue squamous carcinoma cells.^19^ Although HK2 modulates B7‐H3‐induced chemoresistance through the aerobic glycolysis pathway, whether HK2 utilizes EMT and modulates chemoresistance in CRC is still unknown.

In the present study, we demonstrated that Twist1 is required for HK2‐mediated chemoresistance in CRC. Histological and data set analyses revealed that HK2 expression is higher in CRC patients and is related to EMT progression. Moreover, HK2 promoted EMT and oxaliplatin resistance in CRC cells. Importantly, the combination of the HK2 inhibitor 3‐bromopyruvate (3‐bp) and oxaliplatin significantly suppresses tumour growth *in vivo*. HK2 mechanistically stabilized Twist1 from ubiquitination degradation and promoted EMT progression and oxaliplatin resistance in CRC cells. Taken together, our work reveals a previously unrecognized mechanism of HK2 in CRC by promoting EMT and chemoresistance through the regulation of Twist1.

## 2 MATERIALS AND METHODS

### 2.1 Clinical samples

In accordance with the research ethics board guidelines, all CRC patient samples were obtained by approval from The Sixth Affiliated Hospital Sun Yat‐Sen University. Informed written consent was obtained from all patients. All experimental protocols were approved by Sun Yat‐Sen University.

### 2.2 IHC staining and analysis

For immunohistochemistry (IHC) staining, tissue samples were fixed in 4% paraformaldehyde at 4°C for 48 h, embedded in paraffin and sectioned into 5‐μm‐thick slices. The tissue sections underwent a series of processes based on standard protocols, including deparaffinization in xylene, gradient rehydration in different concentrations of alcohol, peroxidase clearing, antigen retrieval at high pressure and blocking by incubation with 3% H_2_O_2_ at room temperature for 10 min. The sections were then subsequently incubated with the following primary antibodies overnight at 4°C: HK2 (Abcam, ab104836), Ki67 (CST, 9449s), TJP1 (Abcam, ab216880), E‐cadherin (CST, 3195), vimentin (CST, 5741) and Twist1 (Abcam, ab175430). Next, immunohistochemistry staining was detected by an ABsin secondary antibody detection kit (ABsin, abs957) according to the manufacturer's protocols. The last step was counterstaining of the cell nuclei with haematoxylin.

For IHC analysis, the sample sections were imaged by light microscopy at 10× and 20× magnification, and five fields of view were selected from each section for analysis. Protein expression was scored according to staining intensity and staining percentage in terms of area. The staining degree of sections was assessed with the following scale: 1, negative; 2, weak staining; 3, moderate staining; or 4, strong staining. The positive staining rate was graded as follows: 1, <25%; 2, 25%–50%; 3, 51%–75%; or 4, 76%–100%. The total scores of each sample were calculated by multiplying the staining degree and staining intensity scores.

### 2.3 Cell culture and reagents

RKO (ATCC CRL‐2577) and HCT8 (ATCC CCL‐244) cells were kindly presented by Professor Yin from Sun Yat‐Sen University School of Pharmaceutical Science. 293T (ATCC CRL‐11268) cells were obtained from ATCC. All cell lines used in this study were cultured in high glucose DMEM (Gibco, C11995500BT) supplement with 10% foetal bovine serum (AusGene, FBSSA500‐S) and 1% Pen‐Strep solution.

### 2.4 Establishment of stable HK2 knockdown cells

HCT8 and RKO control shRNA (con‐shRNA) and HK2‐shRNA cells were established by lentiviral infection using the pLKO.1‐puromycin transfer plasmid (ATCC #8453). The sequences for shRNA against human HK2 are listed below:

HK2‐shRNA1‐F: 5′‐GTAACATTCTCATCGATTTCTCGAGAAATCGATGAGAATGTTAC‐3′.

HK2‐shRNA2‐F: 5′‐AAAGACATCTCAGACATTGCTCGAGCAATGTCTGAGATGTCTTT‐3′. We cotransfected the psPAX2 packaging plasmid and pMD2.G envelope plasmid into 293T cells to produce retrovirus. Viral supernatants were collected after transfection for 48 h. The supernatants were used to infect 5 × 10^5^ HCT8 and RKO cells after filtration. Twenty‐four hours after viral infection, HCT8 and RKO cells were selected with puromycin (InvivoGen, ant‐pr‐1). HK2 knockdown efficiency was detected at both the mRNA and protein levels.

### 2.5 Plasmid construction and transient transfection

The coding sequences of Flag‐ or HA‐tagged human HK2 (NCBI Gene ID: 3099) and Twist1 (NCBI Gene ID: 7291) were amplified by reverse transcription PCR and cloned into the pcDNA3.1(+) vector. All clones were sequenced, and protein expression was confirmed by Western blot. HCT8 and RKO cells were transiently transfected using Lipofectamine™ 3000 (Invitrogen, L3000015) according to the manufacturer's instructions.

### 2.6 Cell proliferation and viability assays

HCT8 and RKO cells (6 × 10^3^ cells per well) were seeded into a 96‐well plate and incubated for 24 h. Cells were treated with oxaliplatin (HCT8: 10, 20, 40, 80 and 150 μM; RKO: 5, 20, 40, 80 and 100 μM) for 48 h. Cell viability was assessed with CCK‐8 kits (A311‐02), and the optical density (OD) at 450 nm was assessed with an EPOCH spectrophotometer (BioTek Instruments).

### 2.7 Cell cycle analysis

HCT8 and RKO cells (2 × 10^5^ cells per well) were seeded into 6‐well plates. HCT8 cells were treated with 3‐bp (25 μM) and/or oxaliplatin (25 μM), and RKO cells were treated with 3‐bp (25 μM) and/or oxaliplatin (25 μM) for 48 h. A total of 1 × 10^6^ cells per sample were stained with Cell Cycle Staining Kits (Multi Sciences, CCS012) and detected by CytoFLEX (Beckman Coulter). The data were plotted, and a curve was fitted using ModFit LT software (PO Box 247).

### 2.8 Cell apoptosis detection

For apoptosis analysis, HCT8 and RKO con‐shRNA and HK2‐shRNA cells were seeded at 1 × 10^5^ cells per well in 12‐well plates. After 24 h, the cells were treated with oxaliplatin (50 μM) for 48 h. HCT8‐WT and RKO‐WT cells were treated with 3‐bp (50 μM) and/or oxaliplatin (50 μM) for 48 h. The ratio of apoptotic cells was measured using an Annexin V‐FITC Apoptosis Detection Kit (Invitrogen) and Annexin V‐APC Apoptosis Detection Kit (Multi Sciences, AT107) according to the manufacturers' protocols. The apoptotic index was examined by CytoFLEX (Beckman Coulter) and analysed using CytExpert software (Beckman Coulter).

### 2.9 Glucose consumption assay

Cells were seeded in triplicate at a density of 3 × 10^5^ cells/well in 6‐well plates and incubated for 24 h. HCT8 and RKO cells were treated with 3‐bp, and culture media were removed after 48 h of incubation for analysis of glucose and lactate levels by a SBA‐40C Biosensor (Biology Institute of the Shandong Academy of Science). Glucose uptake or lactate production was determined according to the concentration difference between the cell culture medium and fresh medium without cells.

### 2.10 Hexokinase activity assay

HCT8 and RKO cells were treated with 3‐bp (50 μM) for 48 h. Endometrial HK activity was detected with the Hexokinase Assay Kit (ab136957) according to the manufacturer's protocol after collection and counting. In a preliminary trial, the optical density at 450 nm in kinetic mode was measured starting at 20 min of incubation, and 60 min was confirmed to be the ideal incubation period. Hexokinase activity (expressed as nmol NADH generated) was calculated according to the manufacturer's instructions.

### 2.11 Western blot and immunoprecipitation assay

Cell lysates for Western blot were collected in RIPA lysis buffer containing protease inhibitor cocktail (Thermo, 78443) for 30 min on ice and centrifuged at 13,200*g* for 10 min at 4°C to obtain supernatant. Protein quantification was performed with the Bradford Protein Assay Kit (Thermo, 23236). For immunoprecipitation of targeted proteins, the supernatants of collected samples were incubated with the corresponding antibody and protein A/G magnetic beads or with anti‐DYDDDDK (MBL, M185‐11) and anti‐HA (MBL, M180‐11) magnetic bead antibodies at 4°C overnight. Then, the beads were separated by a magnetic device. Cell proteins were separated by SDS‐PAGE electrophoresis followed by transfer to PVDF membranes (Millipore, ISEQ00010). After blocking with 5% skim milk (BD Difco, 232100) in Tris‐buffered saline containing 0.5% Tween 20 for 1 h, the membranes were incubated with the corresponding antibodies overnight. The targeted proteins were visualized by an enhanced chemiluminescence (ECL) detection kit (NCM Biotech, P10300) after incubation with horseradish peroxidase‐conjugated antibodies. The primary antibodies included HK2 (Abcam, ab104836), TJP1 (Abcam, ab216880), E‐cadherin (CST, 3195), vimentin (CST, 5741), Twist1 (Abcam, ab175430 and Santa Cruz, sc‐81417), β‐actin (CST, 4970S), anti‐Flag (CST, 14793), anti‐HA (CST, 3724S) and ubiquitin (CST, 3936T).

### 2.12 Immunofluorescence

For immunofluorescence assay, cells were cultured on glass‐bottom dishes. The RKO cells were then fixed with 4% paraformaldehyde and subjected to membrane permeabilization, blocking and antibody incubation overnight. Nuclei were stained with 4,6‐diamidino‐2‐phenylindole (DAPI, Invitrogen, 62248) and captured by a microscope (Eclipse Ti2E, NIKON). The primary antibodies include the following: HK2 (Abcam, ab209847) and Twist1 (Abcam, ab175430).

### 2.13 Reverse transcription‐quantitative PC (RT‐qPCR)

Total RNA was extracted using TRIzol (Invitrogen, 15596018), and 1 μg of total RNA product was reversed transcripted by using the Reversed Transcription Kit (Takara, RR047A) to get cDNA. The RT‐qPCR was performed using SYBR Green reagent (Takara TB Green, RR420A), and the mRNA level was detected by ABI Step‐one Detection System. β‐actin was used as the internal control. The primers in RT‐qPCR assay were as follows: h‐β‐actin: CATGTACGTTGCTATCCAGGC (Forward) and CTCCTTAATGTCACGCACGAT (Reverse); h‐HK2: CCGGAGCATCTCTAACAAGGA (Forward) and GCATCCGCCTCTAGCACAT (Reverse).

### 2.14 Transwell and wound‐healing assays

The Transwell assay was carried out using chambers with filters (pore size of 8 μm) coated with Matrigel. Cell suspensions (1 × 10^5^ cells per well) were seeded into the upper chamber in 200 μl of serum‐free medium. Complete medium was added to the lower chamber. After incubation for 48 h, invasive cells on the bottom surface of the filters were fixed with 4% formaldehyde for 15 min at room temperature and stained with 0.1% crystal violet. For the wound‐healing assay, cells were cultured in 60‐mm plates to reach 90% confluence. The cell monolayer was scratched with a pipette tip, washed three times with PBS to remove the detached cells and incubated in medium containing 10% foetal bovine serum. The scratched areas were photographed at 0 and 48 h. All experiments were performed at least three times.

### 2.15 Xenograft tumorigenicity assay

For the animal assay, RKO cells were subcutaneously injected into 5‐ to 6‐week‐old *BALB*/*c* nude mice. All animal experiments were approved by the Animal Ethics Committee of Sun Yat‐Sen University and performed in accordance with the Animal Care and Use guidelines of Sun Yat‐Sen University. Drug treatment started when the tumours reached 100 mm^3^ in size. 3‐bp (1.8 mg/kg) and oxaliplatin (10 mg/kg) were injected intraperitoneally every 3 days. The tumour size and weight of the mice were measured before drug injection every time. Subcutaneous tumour xenografts were removed and conserved for subsequent analysis.

### 2.16 Statistical analysis

For data analysis, we used GraphPad Prism 8 software (San Diego, 265 California) and performed two‐tailed *t* tests and two‐way analysis of variance (ANOVA) to analyse the statistically significant differences. The correlation among the groups for clinical gene expression was determined by two‐sided Pearson chi‐square test analysis. All results are presented as the mean ± standard error of the mean (SEM) from at least three independent experiments. *0.01 < *p* < 0.05; **0.001 < *p *< 0.01; ***p < 0.001.

## 3 RESULTS

### 3.1 HK2 expression is increased in CRC tumour tissues

**FIGURE 1:**
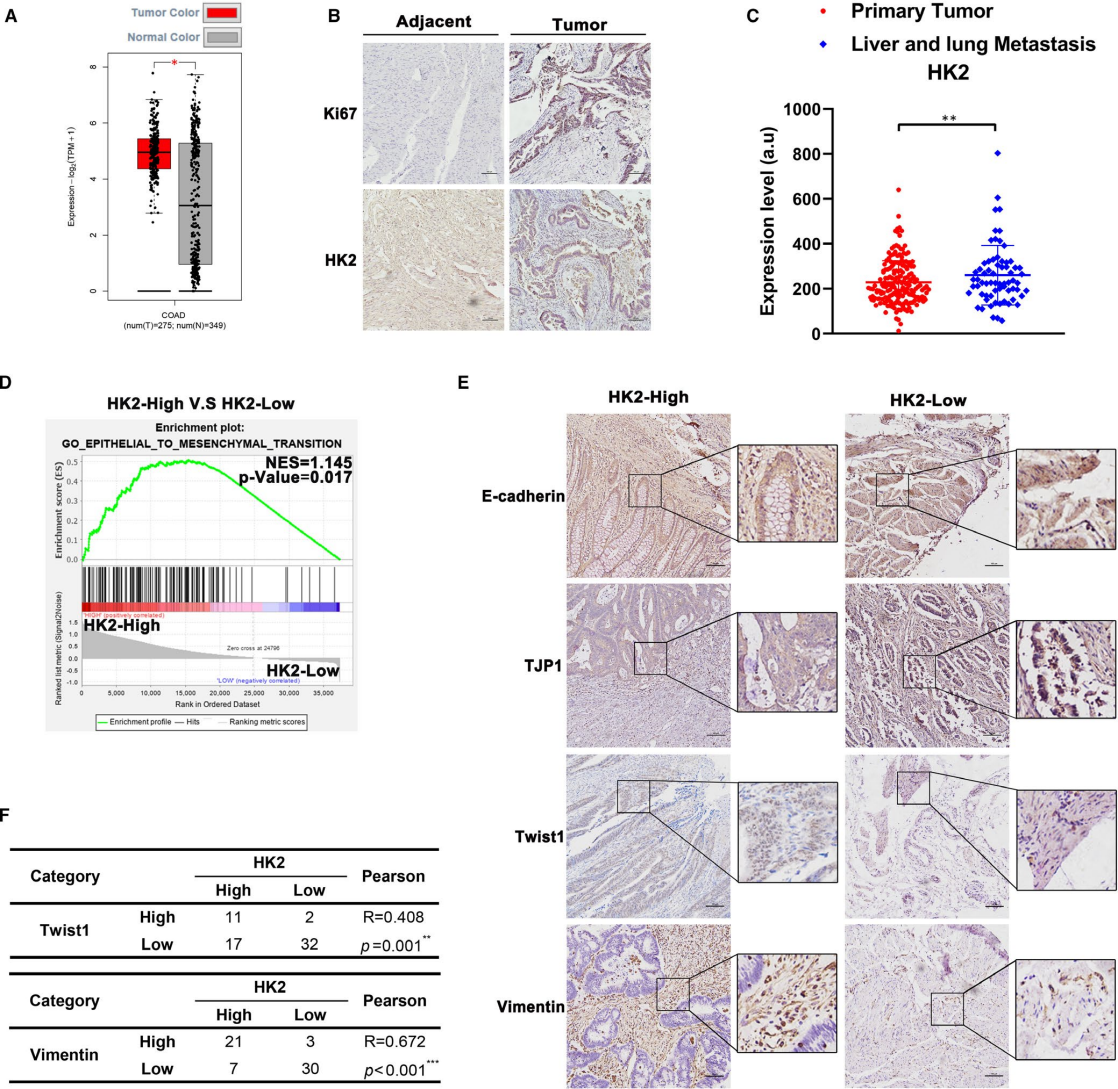
The expression of HK2 increased in CRC patients. (A) The expression of HK2 between normal and tumour tissues in TCGA. (B) HK2 and Ki67 expression in tumour tissues and the corresponding adjacent tissues of CRC by IHC staining. Scale bar = 100 μm. (C) Comparison of the expression of HK2 between primary colorectal tumour tissues and live and lung metastasis colorectal tissues. (D) GSEA was performed to detect enrichment of EMT‐like pathways between HK2‐high and HK2‐low expression cohorts from TCGA. (E) IHC staining of TJP1, E‐cadherin, vimentin and Twist1 in HK2‐high and HK2‐low CRC patient groups. Scale bar = 100 μm. (F) Comparison of high and low HK2 patients with Twist1 (*p* = 0.001) and vimentin (*p* < 0.001) staining by immunohistochemistry. *p < 0.05, **0.001 < *p* < 0.01; ***p < 0.001. Data are represented as the mean ± SEM

To determine whether HK2 expression was different between healthy people and CRC patients, we analysed the clinical COAD dataset from TCGA. Notably, we found that HK2 was highly expressed in CRC patient samples compared with healthy samples (Figure 1A). To further confirm that HK2 exerts oncogenic functions and promotes CRC progression, we compared the overall survival of CRC patients with high vs. low HK2 expression. The results showed that the overall survival rate of patients with high HK2 expression was obviously lower than that of patients with low HK2 expression (Figure S1A). We also performed IHC staining to detect the expression of HK2 and Ki67 in CRCs and the corresponding adjacent tissues in 63 pairs of clinical samples. Staining indicated the upregulation of HK2 in CRC tumour tissues (Figure 1B). Additionally, we analysed the clinical characteristics of CRC patients and found that HK2 was positively correlated with lymphatic invasion (Table S1). Furthermore, we compared HK2 expression between 186 primary colorectal tumour tissues and 67 live and lung metastatic CRC tissues from the clinical COAD data of the Gene Expression Omnibus (GEO) data sets (GSE41258). The results showed that the expression of HK2 in metastatic cancer tissues was higher than that in healthy tissues (Figure 1C). We also utilized TCGA for gene set enrichment analysis (GSEA) to determine which gene set was correlated with the HK2 expression of patients. Interestingly, the mRNA signature of CRC patients with high HK2 expression was most significantly enriched in EMT‐like genes (Figure 1D). The clinical COAD data also revealed a positive correlation between the expression of HK2 and vimentin (Figure S1B). In addition, to confirm that HK2 can regulate EMT in CRC, we performed IHC staining to detect TJP1, E‐cadherin, vimentin and Twist1 expression in the tumour tissues of CRC patient samples (Figure 1E), and the immunohistochemical staining of these proteins exhibited different degrees of positivity (Figure S1C). The results indicated that the expression of Twist1 and vimentin was positively correlated with the HK2 level (Figure 1F). Overall, these findings indicate that HK2 expression in tumour tissues is higher than that in adjacent healthy tissues of CRC patients and is also related to EMT and drug resistance processes.

### 3.2 HK2 knockdown or activity inhibition impairs EMT in CRC cells

**FIGURE 2:**
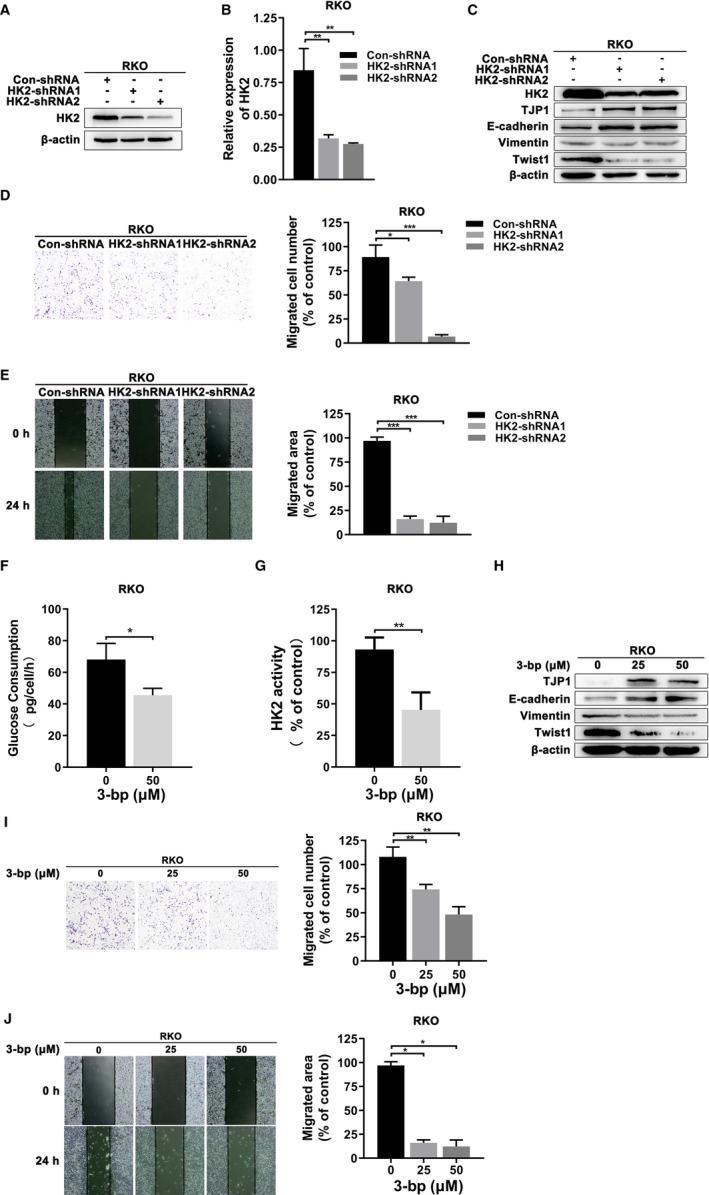
HK2 induced EMT characteristics in CRC cells. (A) Knockdown efficiency of HK2‐shRNAs was analysed by Western blot in RKO. (B) Knockdown efficiency of HK2‐shRNAs was analysed by real‐time PCR in RKO. (C) EMT‐related TJP1, E‐cadherin, vimentin and Twist1 expression was analysed in HK2 knockdown RKO cells by Western blot. (D) Transwell assays were performed to detect the migration ability of RKO after HK2 knockdown. The histogram represents the quantification analysis. (E) Wound‐healing assays were performed to detect the migration ability of the indicated cells. Cells were imaged at 0 and 24 h. The migration distance was analysed by ImageJ. The histogram represents the quantitative analysis. (F) Cellular glucose consumption was detected by a glucose (HK) kit in RKO after treatment with 3‐bp (50 μM) for 48 h at the indicated concentration. (G) A hexokinase assay was performed to measure endometrial HK activity in RKO after treatment with 3‐bp (50 μM) for 48 h. (H) After 3‐bp (25 μM and 50 μM) treatment for 48 h, EMT‐related TJP1, E‐cadherin, vimentin and Twist1 expression was analysed in RKO cells by Western blot. (I) Transwell assays were performed to assess the migration ability of RKO after treatment with 3‐bp (25 μM and 50 μM). The histogram represents quantitative analysis. (J) Wound‐healing assays were performed to assess the migration ability of the indicated cells after treatment with 3‐bp at different concentrations (25 and 50 μM). Cells were imaged at 0 and 24 h. The migration distance was analysed by ImageJ. The histogram represents the quantitative analysis. *p < 0.05; **0.001 < *p* < 0.01; ***p < 0.001. Data are represented as the mean ± SEM

To further verify this assumption, we generated two CRC cell lines (HCT8 and RKO) that had significant efficiency of stable knockdown of HK2 expression (Figure 2A,B and Figure S2A,B). TJP1 and E‐cadherin are epithelial markers, while vimentin and Twist1 are mesenchymal markers in EMT. The results mentioned above showed that knockdown of HK2 decreased vimentin and Twist1 expression and increased the levels of TJP1 and E‐cadherin in CRC cells (Figure 2C and Figure S2C). Given that EMT is a factor in tumour metastasis, we further investigated the migration ability of CRC cells when HK2 was knocked down by Transwell and wound‐healing assays. The results showed that HK2 deficiency decreased the migration ability of CRC cells (Figure 2D,E and Figure S2D,E). Moreover, previous studies have demonstrated that 3‐bp, as an inhibitor of HK2, can separate HK2 from a mitochondrial complex.^20^ We further detected cell glucose consumption in CRC cells after treatment with 3‐bp at several different concentrations. We found that 3‐bp effectively impaired HCT8 and RKO glucose consumption at 50 μM (Figure 2F and Figure S2F) To further understand the function of 3‐bp in CRC cells, we detected HK activity with a hexokinase activity detection kit after treatment with 3‐bp. Likewise, 3‐bp exerted the same inhibitory effect on HK activity in CRC cells (Figure 2G and Figure S2G). We performed Western blot to detect the expression of TJP1, E‐cadherin, vimentin and Twist1 after treatment with two different concentrations of 3‐bp for 48 h. Similarly, EMT molecular expression was downregulated in a concentration‐dependent manner (Figure 2H and Figure S2H). Moreover, the Transwell and wound‐healing assays showed that the migration ability of CRC cells was inhibited after treatment with 3‐bp (Figure 2I,2J and Figure S2I,J). Collectively, these observations suggested that inhibition of HK2 activity or knockdown of HK2 impaired the EMT process in CRC cells.

### 3.3 HK2 regulates oxaliplatin resistance in CRC cells

**FIGURE 3:**
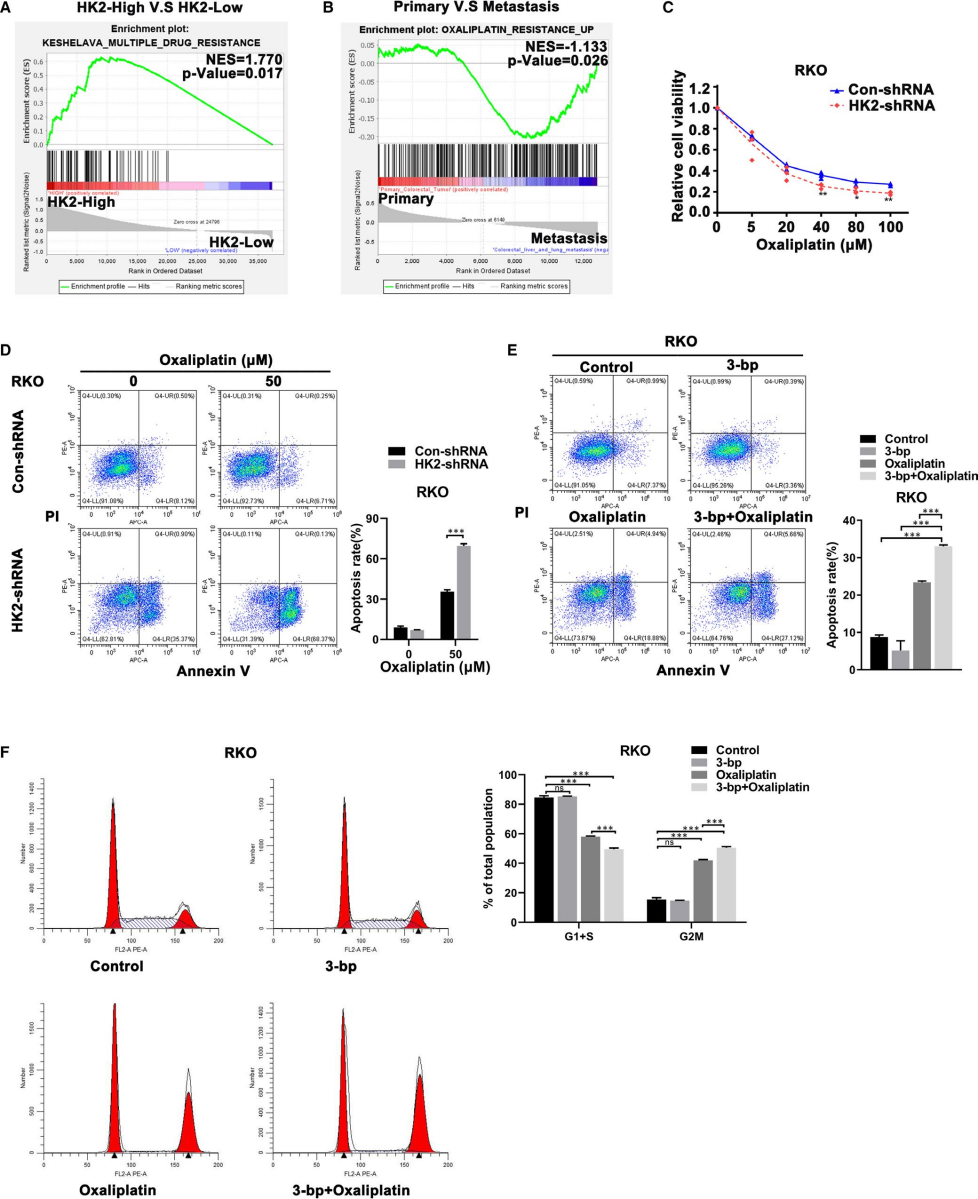
HK2 regulated the effect of oxaliplatin in CRC cells. (A) GSEA was performed to detect enrichment of drug resistance‐like pathways between HK2‐high and HK2‐low expression cohorts from TCGA. (B) GSEA was performed to detect enrichment of oxaliplatin resistance‐like pathways between primary colorectal tumours and liver and lung metastases. (C) Cell viability of con‐shRNA and HK2‐shRNA RKO cells was assessed after oxaliplatin treatment at the indicated concentrations. (D) Flow cytometry assays were performed to analyse the apoptotic rate in con‐shRNA and HK2‐shRNA RKO cells after oxaliplatin (50 μM) treatment. (E) The apoptotic rate was analysed after 3‐bp (50 μM) and/or oxaliplatin (50 μM) treatment and compared with that of untreated cells for 48 h by flow cytometry of the indicated cells. (F) Cell cycle analysis was performed after 3‐bp (25 μM) and/or oxaliplatin (25 μM) treatment and compared with that of untreated cells for 48 h by flow cytometry in the indicated cells. The histogram represents the quantitative analysis. *p < 0.05; **0.001 < *p* < 0.01; ***p < 0.001. Data are represented as the mean ± SEM

Oxaliplatin is the first‐line drug in CRC chemotherapy, and we further explored whether HK2 regulates oxaliplatin reactivity in CRC. Similarly, we discovered that the mRNA signature of CRC patients with high HK2 expression from TCGA was most significantly enriched in drug resistance‐like genes (Figure 3A). In addition, we obtained a gene set that included the top 626 genes that were upregulated in oxaliplatin‐resistant CRC cells by analysing GEO data sets (GSE77932). The mRNA signature of live and lung metastatic CRC tissues was significantly enriched in oxaliplatin resistance upregulated genes (Figure 3B). We wanted to determine whether HK2 regulated oxaliplatin resistance in CRC cells by increasing the development of EMT. Therefore, we detected cell viability in HK2 knockdown CRC cells using a Cell Counting Kit‐8 (CCK‐8) assay (Figure 3C and Figure S3A). The results showed that HK2 knockdown significantly increased the sensitivity of CRC cells to oxaliplatin. Next, we performed a flow cytometry assay to confirm that HK2 knockdown also increased the proapoptotic response of CRC cells to oxaliplatin treatment (Figure 3D and Figure S3B). Similarly, when we detected the apoptotic rate after treatment with 3‐bp and/or oxaliplatin, 3‐bp dramatically enhanced the proapoptotic effect of oxaliplatin in CRC cells. However, the induction of CRC cell apoptosis by 3‐bp alone was not significantly different from that in the control group (Figure 3E and Figure S3C). As a cell cycle blocker, oxaliplatin can induce DNA interstrand crosslinks and DNA‐protein crosslinks that cause DNA lesions and cell cycle arrest.^21^ Thus, we analysed the change in the cell cycle of HK2 knockdown CRC cells via the flow cytometry assay after treatment with 3‐bp and/or oxaliplatin as indicated above. Similarly, there was no significant difference between the control and 3‐bp groups. However, after oxaliplatin treatment, the number of G2/M‐phase cells increased compared with that in the control and 3‐bp groups. After treatment with the combination of 3‐bp and oxaliplatin, the proportion of cells in G2/M‐phase was higher than that in the other phases (Figure 3F and Figure S3D). In general, we concluded that HK2 regulates oxaliplatin resistance and that HK2 inhibition enhances oxaliplatin sensitivity in CRC cells.

### 3.4 HK2 interacts with Twist1 to stabilize Twist1 in CRC cells

Since we found that HK2 regulated EMT by influencing relevant marker expression, we assumed that EMT transcription factors participated in signal transduction in the cells.

**FIGURE 4:**
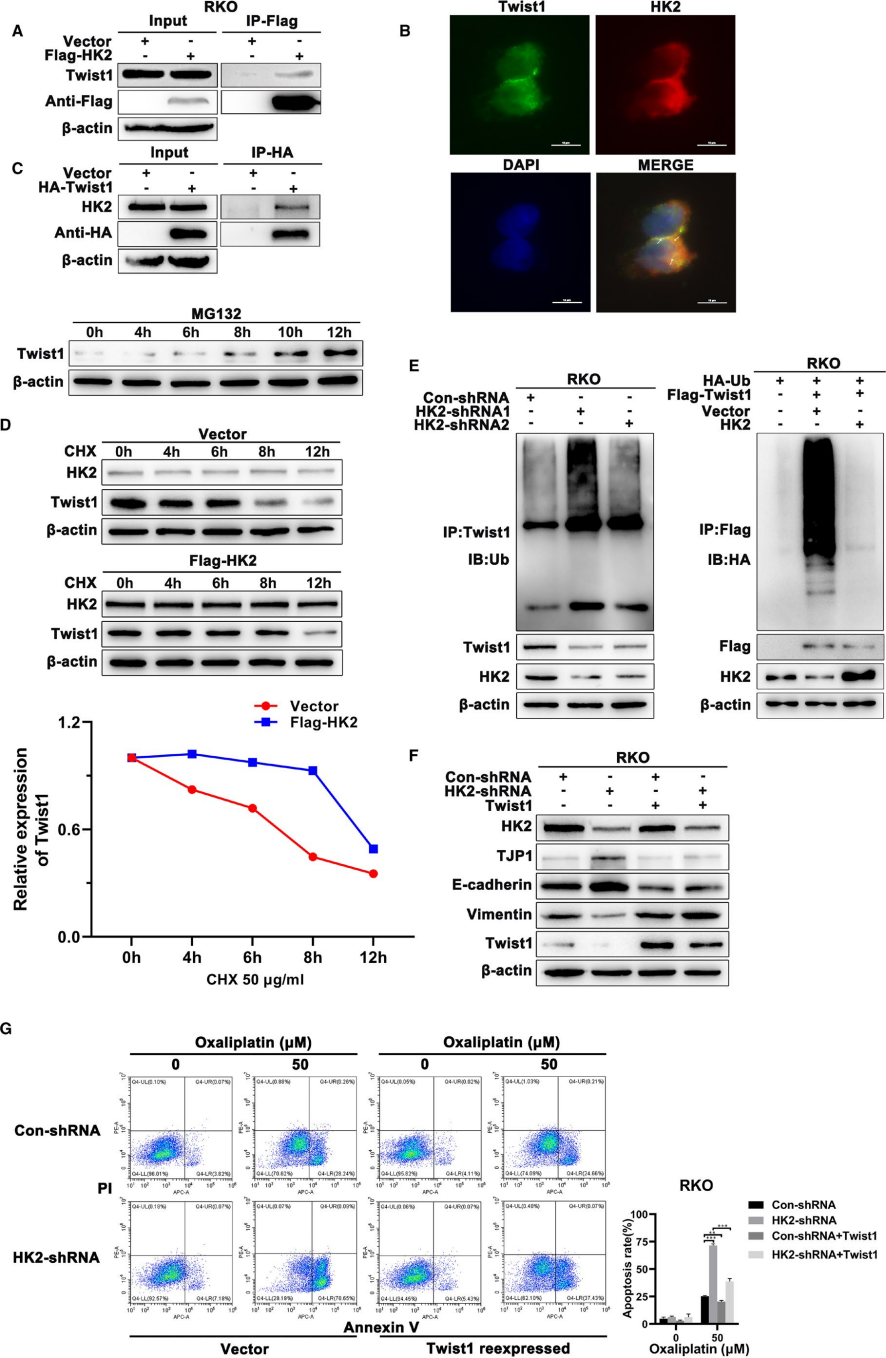
HK2 regulated EMT and oxaliplatin resistance via Twist1 in CRC cells. (A) RKO cells were transfected with Flag‐tagged HK2 plasmid or HA‐tagged Twist1 for 48 h and then lysed and analysed by immunoprecipitation using anti‐Flag or anti‐HA magnet beads, followed by immunoblotting with the indicated antibody. (B) Immunofluorescence was performed to detect HK2 and Twist1 in RKO cells. Scale bar = 25 μm. (C) RKO cells were treated with 100 μM MG132 for 4, 6, 8, 10 and 12 h, and Western blot was performed to detect the expression of Twist1. (D) Western blot was performed to detect the expression of Twist1 after treatment with HK2 after CHX (50 μg/ml) for 4, 6, 8 and 12 h in RKO cells. (E) Coimmunoprecipitation was performed to detect endogenous ubiquitin levels in HK2 knockdown RKO cells by using anti‐Twist1 antibody, and RKO cells were cotransfected with the indicated plasmids and then lysed and analysed by immunoprecipitation using anti‐Flag magnet beads followed by immunoblotting with anti‐Flag or anti‐HA antibody. (F) HK2 knockdown RKO cells were transfected with Twist1 or control plasmid for 24 h and collected for cell lysates. The expression levels of EMT‐related TJP1, E‐cadherin, vimentin, and Twist1 were determined by Western blot. (G) HK2 knockdown RKO cells were transfected with Twist1 or control plasmid for 24 h and treated with oxaliplatin (50 μM) for 48 h. Flow cytometry was performed to analyse the apoptotic rate in the indicated RKO cells. **0.001 < *p* < 0.01; ***p < 0.001. Data are represented as the mean ± SEM

Interestingly, we performed coimmunoprecipitation and immunofluorescence assays by transfecting cells with HK2‐FLAG or Twist1‐HA plasmids and demonstrated the interaction between HK2 and Twist1 in CRC cells (Figure 4A). Moreover, immunofluorescence assays showed the colocalization of the two proteins in RKO cells (Figure 4B). To further define the function of the HK2 and Twist1 interaction, we detected Twist1 expression in RKO cells treated with MG132, and Twist1 degradation was significantly blocked (Figure 4C). The results showed that Twist1 degradation occurs through the ubiquitination pathway. After treating cells with cycloheximide (CHX) to inhibit protein translation, the Twist1 degradation rate was suppressed in HK2‐overexpressing CRC cells (Figure 4D). Moreover, a coimmunoprecipitation assay showed that the expression of HK2 dramatically removed the polyubiquitin of Twist1. The results indicated that the interaction between HK2 and Twist1 may prevent Twist1 from proteasome degradation (Figure 4E). Furthermore, we sought to determine whether Twist1 expression regulated by HK2 caused oxaliplatin resistance. We overexpressed Twist1 in HK2 knockdown and control CRC cells and then assessed the expression of EMT‐related markers and the sensitivity of cells to oxaliplatin treatment. As expected, TJP1 and E‐cadherin were significantly downregulated after Twist1 expression, while vimentin was upregulated when Twist1 was re‐expressed (Figure 4F). Flow cytometry assay results showed that the apoptotic rate was decreased in HK2 knockdown cells overexpressing Twist1 (Figure 4G). Collectively, these results indicated that HK2 interacts with Twist1 to regulate EMT and oxaliplatin resistance in CRC cells.

### 3.5 The combination of 3‐bp and oxaliplatin significantly suppresses tumorigenesis and EMT development *in vivo*

**FIGURE 5:**
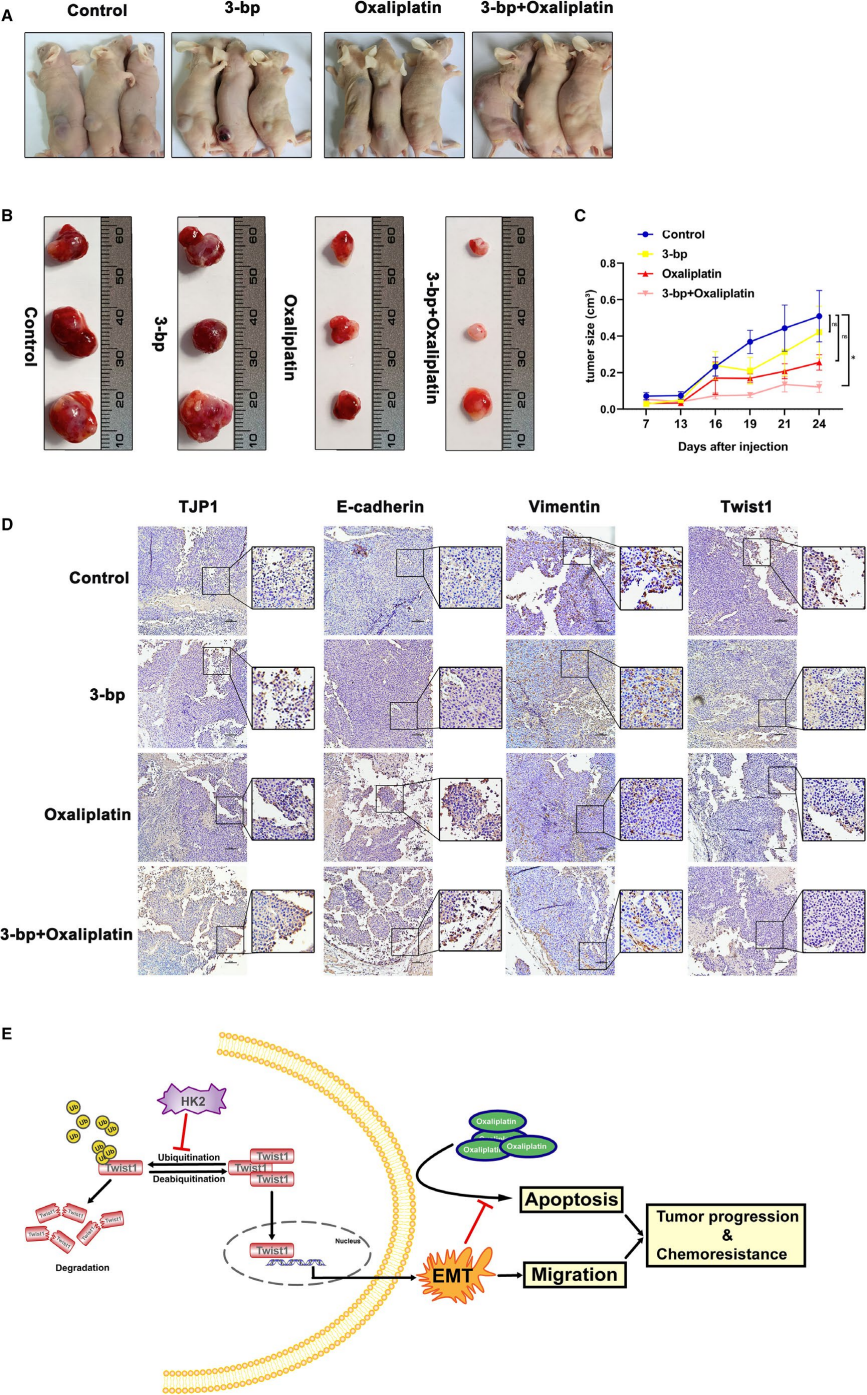
The combination of 3‐bp and oxaliplatin significantly suppressed tumorigenesis and EMT development in vivo. (A) Macroscopic images of xenograft mice at day 24. (B) Representative gross samples retrieved from xenograft nude mice. The tumours were resected and measured as shown. (C) After RKO cell infection for 7 days, different groups were treated with 3‐bp (1.8 mg/kg), oxaliplatin (10 mg/kg) or a combination of the two drugs intraperitoneally every 3 days, and the tumour volumes were measured and recorded. (D) IHC staining of EMT‐related E‐cadherin, vimentin, Twist1 and TJP1 expression in sections from xenograft mice with the indicated treatment. Scale bar = 100 μm. (E) Illustrative model showing the proposed mechanism by which HK2 promotes EMT and induces chemoresistance in CRC by interacting with Twist1. *p < 0.05. Data are represented as the mean ± SEM

According to the aforementioned findings, we wondered whether inhibition of HK2 activity could enhance CRC sensitivity after oxaliplatin treatment. To define the combined effect of 3‐bp and oxaliplatin, we constructed a subcutaneous CRC mouse model. After 1 week, we divided the mice into four groups and treated them with 3‐bp, oxaliplatin and a combination of two drugs intraperitoneally every 3 days and measured the tumour size in each treatment group. We found that the combination treatment significantly inhibited tumour growth (Figure 5A–C). In addition, IHC staining of TJP1, E‐cadherin, vimentin and Twist1 revealed that the combination of 3‐bp and oxaliplatin dramatically suppressed the development of EMT *in vivo* (Figure 5D).

## 4 DISCUSSION

The Warburg effect, whereby more aerobic glycolysis occurs in tumour cells than in normal cells, is one of the hallmarks of cancer.^4^ In this study, we found that high HK2 expression is related to EMT progression and drug resistance in samples from the COAD cohort of TCGA. Subsequently, we demonstrated that HK2 increased mesenchymal‐like protein expression and decreased oxaliplatin sensitivity by interacting with Twist1 in CRC cells. The combination of the HK2 inhibitor 3‐bp and oxaliplatin effectively decreased tumorigenesis compared with oxaliplatin treatment alone. Our study revealed an important association between aerobic glycolysis and chemoresistance via HK2, suggesting a novel target for CRC chemotherapy.

Given that database analysis showed EMT and multi‐drug resistance enrichment in the CRC cohort with high HK2 expression, we hypothesized that HK2 simultaneously modulated EMT and chemoresistance in CRC, which has not yet been reported. Previous research has reported that miR‐125‐5p decreases the cellular glycolytic rate and increases cisplatin sensitivity by directly targeting the 3′ untranslated region (UTR) of HK2 in CRC.^22^ B7‐H3, an immune checkpoint molecule, induces CRC chemoresistance by promoting HK2 expression to increase glucose consumption and lactate production.^23^ Consistently, in our study, knockdown of HK2 expression or inhibition of HK2 activity reduced glucose consumption and decreased oxaliplatin‐dependent apoptosis. Moreover, either treatment significantly suppressed EMT‐related protein marker expression *in vitro* or *in vivo*. Notably, investigations showed that upregulation of glucose metabolism, especially HK2 expression, was associated with the chemoresistance phenotypes of breast cancer cells.^24^, ^25^

Oxaliplatin is a third‐generation platinum drug that is used for the treatment of multiple cancers. In 2000, oxaliplatin was first introduced for the treatment of metastatic CRC, in which cisplatin and carboplatin had been demonstrated to be ineffective.^26^ Copper transporters, the solute carrier superfamily of membrane transporters,^27^ ABC transporters^28^ and the glutathione system^29^ have been reported to be associated with oxaliplatin resistance. In addition, tumour cells utilize multiple cell signalling pathways to escape oxaliplatin‐induced apoptosis, necrosis and autophagy. While the loss of proapoptotic Bax decreases sensitivity to oxaliplatin,^30^ downregulation of the antiapoptotic members Bcl‐2 and Bcl‐xL increases sensitivity to oxaliplatin.^31^ Although previous studies have suggested multiple roles for HK2 in cancer cell chemoresistance, we demonstrated that HK2 mediates oxaliplatin resistance by stabilizing Twist1. In addition, Twist1 was found to be upregulated via the EZH2‐SLFN11 axis during the development of resistance to a variety of agents in small‐cell lung cancer.^32^ Twist1‐mediated activation of E2F4‐RBL2 and inhibition of EP300 have been shown to regulate the DNA damage response in autophagy deficiency.^33^ Mechanistically, oxaliplatin functions as a DNA‐interacting agent, disrupting DNA replication and transcription.^34^ The nucleotide excision repair pathway has been described to be the main oxaliplatin‐induced damage repair system.^35^ Our experiments provide further support that oxaliplatin treatment increases Twist1 transcriptional levels, but the potential targets of Twist1 modulation in CRC oxaliplatin resistance remain to be assessed in our future research.

Epithelial‐mesenchymal transition progression is dispensable for metastasis but required for breast and pancreatic cancer chemoresistance.^36^, ^37^ In our study, Twist1 reversed HK2 knockdown‐induced oxaliplatin sensitivity and EMT retardation. Although inhibition of HK2 plus oxaliplatin effectively decreased tumorigenesis in vivo, there was not enough proof about the long‐term metastasis regulated by HK2. Nevertheless, it is reported that Twist1 can increase the expression of HK2 as well as several other glycolytic genes and participates in glycolytic pathways.^38^ Analogously, EMT‐related proteins participating in chemoresistance have been found in ATM‐ZEB1 feedback in breast cancer.^13^, ^39^ FBXW7 directly binds and degrades the EMT‐inducing transcription factor ZEB2 and impairs cell migration and chemoresistance.^17^ Thus, we suppose that HK2 and Twist1 may be feedback genetic modifiers of drug resistance that can be targeted for therapy.

In summary, we explored the relationship between EMT and drug resistance‐mediated by HK2. Our results demonstrated that HK2 knockdown enhances oxaliplatin‐induced apoptosis in CRC cells via an increase in ubiquitin‐mediated degradation of Twist1 (Figure 5E). Therefore, we hypothesize that CRC cells may alter metabolism to promote EMT by forming a positive feedback loop between HK2 and Twist1 to escape the apoptosis induced by oxaliplatin. Nevertheless, the combination of 3‐bp and oxaliplatin may constitute a treatment for HK2‐dependent oxaliplatin‐resistant CRC.

## CONFLICT OF INTEREST

The authors have no conflict of interest to declare.

## AUTHOR CONTRIBUTIONS

**Bo Zhang:** Investigation (equal); Project administration (equal); Writing‐original draft (equal). **Sze‐Hoi Chan:** Investigation (equal); Project administration (equal); Writing‐original draft (equal). **Xue‐Qi Liu:** Investigation (equal); Validation (equal). **Yuan‐Yuan Shi:** Investigation (equal); Validation (equal). **Zhao‐Xia Dong:** Investigation (equal); Validation (equal). **Xin‐Rong Shao:** Investigation (equal); Validation (equal). **Li‐Yuan Zheng:** Data curation (equal); Validation (equal). **Zhi‐Ying Mai:** Data curation (equal); Validation (equal). **Tian‐Liang Fang:** Data curation (equal); Validation (equal). **Li‐Zhi Deng:** Data curation (equal); Supervision (equal). **Di‐Sheng Zhou:** Data curation (equal); Validation (equal). **Shu‐Na Chen:** Conceptualization (equal); Project administration (equal); Writing‐review & editing (equal). **Miao Li:** Conceptualization (equal); Project administration (equal); Writing‐review & editing (equal). **Xing‐Ding Zhang:** Conceptualization (equal); Project administration (equal); Writing‐review & editing (equal).

## Supporting information

Fig S1‐3

Table S1

## Data Availability

The data that support the findings of this study are available from the corresponding author upon reasonable request.
