# Supplementary material for: Targeting hexokinase 2 increases the sensitivity of oxaliplatin by Twist1 in colorectal cancer
Source: J Cell Mol Med. 2021 Aug 10;25(18):8836–49. doi: 10.1111/jcmm.16842 (PMC8435428; doi:10.1111/jcmm.16842)
Supplement: Supplementary file 1 — Fig S1‐3 [file JCMM-25-8836-s002.docx]

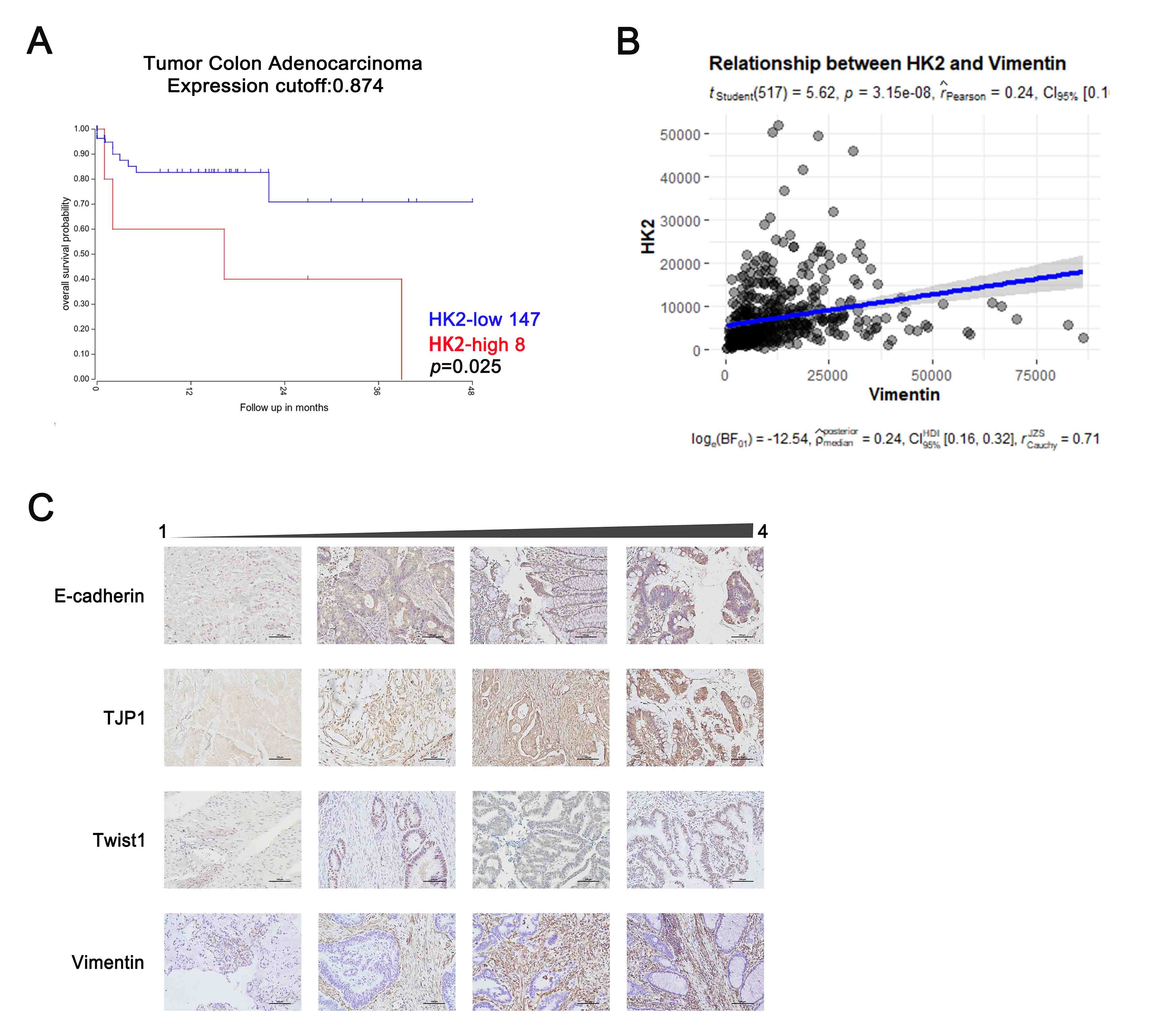


**Supplemental Figure 1.** The expression of HK2 increased in CRC patients.

A, Kaplan–Meier analysis (https://hgserver1.amc.nl/cgi-bin/r2/main.cgi) of overall survival rate of low (blue line) and high (red line) HK2 expression for colorectal cancer patients in TCGA database. *, p< 0.05. B, The correlation between the expression of HK2 and Vimentin in clinical COAD dataset form TCGA database. ***, p< 0.001. C, The different positive immunohistochemical staining degrees of EMT related marker proteins. **
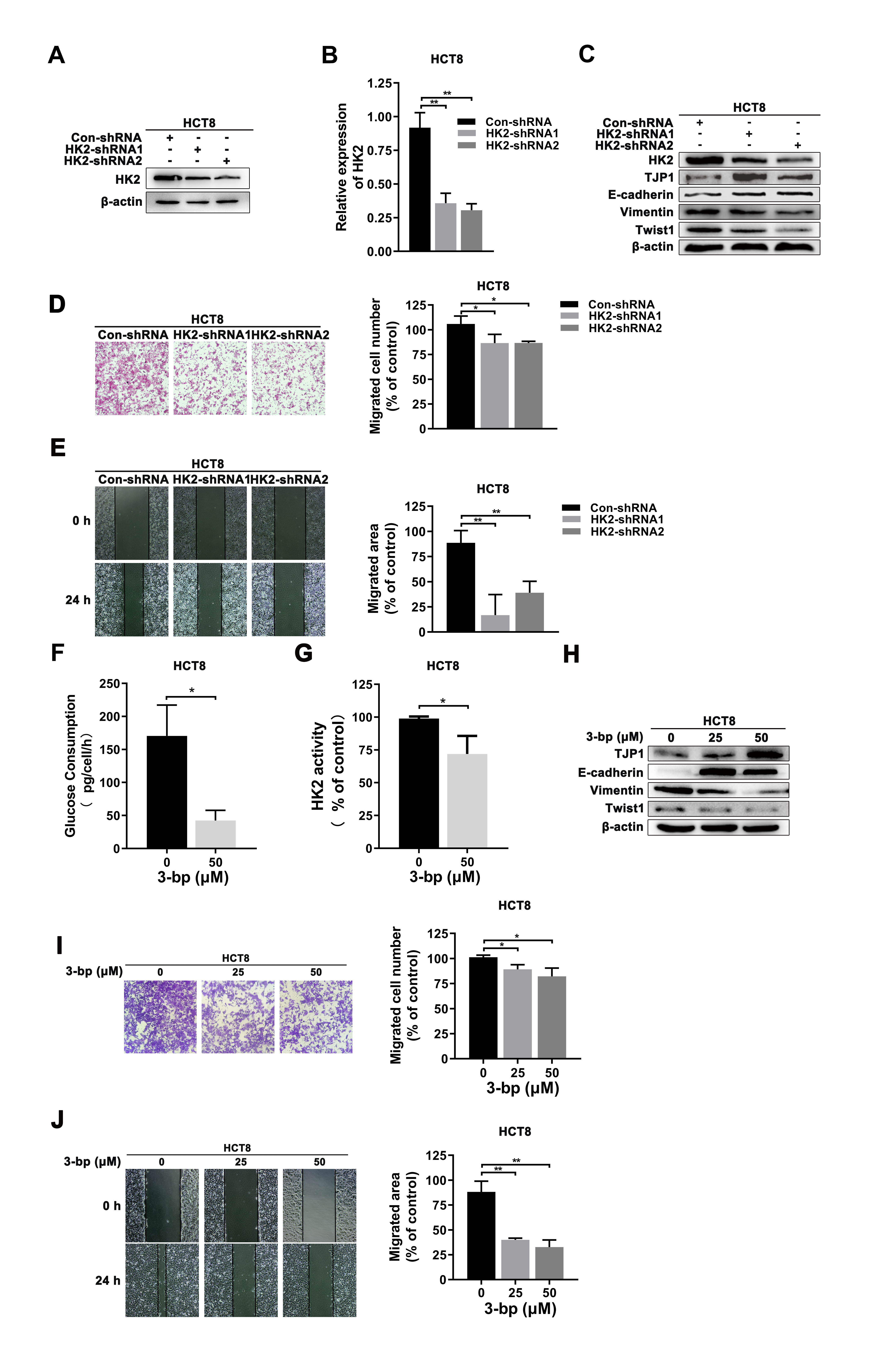
**

**Supplemental Figure 2.** HK2 induced EMT characteristics in CRC cells.

A, Knockdown efficiency of HK2-shRNAs was analyzed by western blotting in HCT8. B, Knockdown efficiency of HK2-shRNAs was analyzed by real-time PCR in HCT8. C, EMT-related TJP1, E-cadherin, Vimentin, and Twist1 expression was analyzed in HK2-knockdown HCT8 by western blotting. D, Transwell assays were performed to detect the migration ability of HCT8 after HK2 knockdown. The histogram represents the quantification analysis. *, p < 0.05. Data are represented as mean ± SEM. E, Wound healing assays were performed to detect the migration ability of the HCT8. Cells were imaged at 0 hours and 24 hours. The migration distance was analyzed by ImageJ. The histogram represents the quantification analysis. **, 0.001< p <0.01. Data are represented as mean ± SEM. F, Cell glucose consumption was detected by a glucose (HK) kit in HCT8 after treatment with 3-bp (50 μM) for 48 hours at the indicated concentration. *, p < 0.05. Data are represented as mean ± SEM. G, A hexokinase assay was performed to measure endometrial HK activity in HCT8 after treatment with 3-bp (50 μM) for 48 hours. *, p < 0.05. Data are represented as mean ± SEM. H, After 3-bp (25 μM and 50 μM) treatment for 48 hours, EMT-related TJP1, E-cadherin, Vimentin, and Twist1 expression was analyzed in HCT8 by western blotting. I, Transwell assays were performed to detect the migration ability of HCT8 after treatment with 3-bp (25 μM and 50 μM). The histogram represents quantification analysis. *, p < 0.05. Data are represented as mean ± SEM. J, Wound healing assays were performed to detect the migration ability of the HCT8 after treatment with 3-bp at different concentrations (25 μM and 50 μM). Cells were imaged at 0 hours and 24 hours. The migration distance was analyzed by ImageJ. The histogram represents the quantification analysis. **, 0.001< p <0.01. Data are represented as mean ± SEM. **
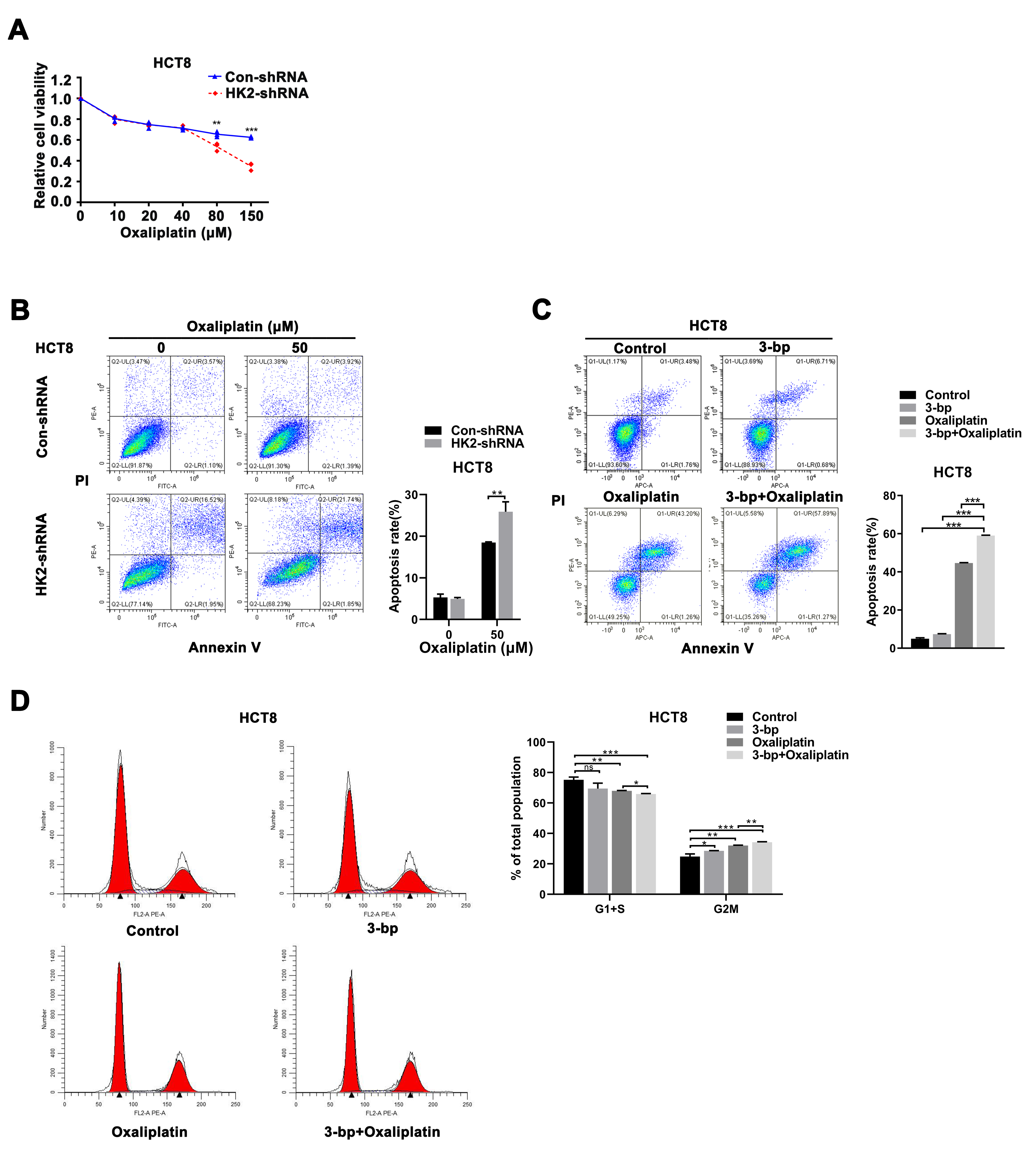
**

**Supplemental Figure 3**. HK2 regulated the effect of oxaliplatin in CRC cells.

A, Cell viability of con-shRNA and HK2-shRNA HCT8 was detected after oxaliplatin treatment at the indicated concentrations. *, p < 0.05; **, 0.001< p < 0.01; ***, p < 0.001. Data are represented as mean ± SEM. B, Flow cytometry assays were performed to analyze the apoptotic rate in con-shRNA and HK2-shRNA HCT8 after oxaliplatin (50 μM) treatment. **, 0.001< p < 0.01; ***, p < 0.001. Data are represented as mean ± SEM. C, The apoptotic rate was analyzed after 3-bp (50 μM) and/or oxaliplatin (50 μM) treatment in comparison to untreated cells for 48 hours by flow cytometry in the indicated cells. ***, p < 0.001. Data are represented as mean ± SEM. D, Cell cycle analysis was performed after 3-bp (25 μM) and/or oxaliplatin (25 μM) treatment in comparison to untreated cells for 48 hours by flow cytometry in the indicated cells. The histogram represents the quantification analysis. *, p < 0.05; **, 0.001< p <0.01; ***, p < 0.001. Data are represented as mean ± SEM.
