# Supplementary material for: Targeting hexokinase 2 increases the sensitivity of oxaliplatin by Twist1 in colorectal cancer
Source: J Cell Mol Med. 2021 Aug 10;25(18):8836–49. doi: 10.1111/jcmm.16842 (PMC8435428; doi:10.1111/jcmm.16842)
Supplement: Supplementary file 2 — Table S1 [file JCMM-25-8836-s001.docx]

**Supplemental Table 1: The correlation between HK2 expression and clinical characteristics of CRC patients.**

**
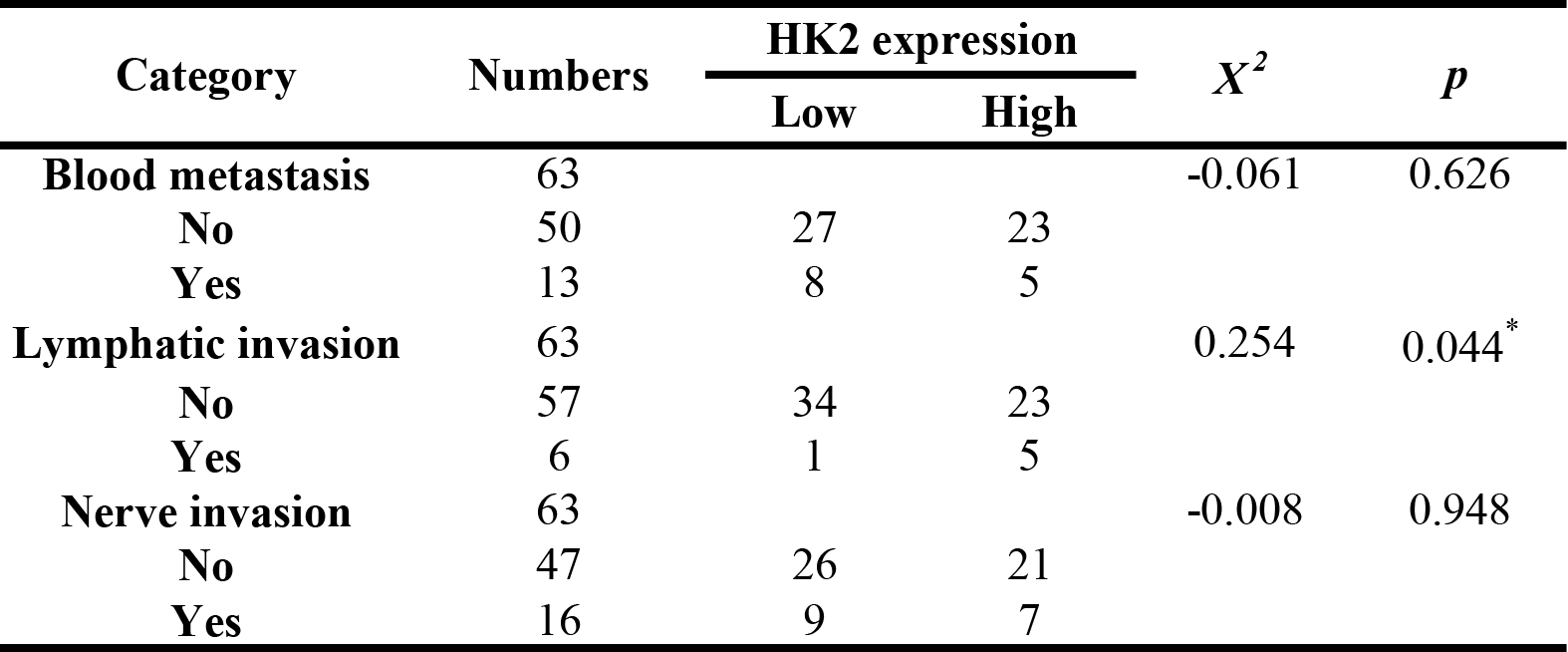
**
